# Towards a leptospirosis early warning system in northeastern Argentina

**DOI:** 10.1098/rsif.2023.0069

**Published:** 2023-05-17

**Authors:** Martín Lotto Batista, Eleanor M. Rees, Andrea Gómez, Soledad López, Stefanie Castell, Adam J. Kucharski, Stéphane Ghozzi, Gabriela V. Müller, Rachel Lowe

**Affiliations:** ^1^ Department for Epidemiology, Helmholtz Centre for Infection Research, 38124 Brunswick, Germany; ^2^ Barcelona Supercomputing Center (BSC), 08034 Barcelona, Spain; ^3^ Centre for Mathematical Modelling of Infectious Diseases, London School of Hygiene and Tropical Medicine, London WC1E 7HT, UK; ^4^ Centre on Climate Change and Planetary Health, London School of Hygiene and Tropical Medicine, London WC1E 7HT, UK; ^5^ Centre for Studies of Climate Variability and Climate Change (CEVARCAM), National University of Litoral (UNL), S3000 Santa Fe, Argentina; ^6^ National Council for Scientific and Technical Research (CONICET), C1425FQB Santa Fe, Argentina; ^7^ Catalan Institution for Research and Advanced Studies (ICREA), 08010 Barcelona, Spain

**Keywords:** leptospirosis, climate, El Niño, Bayesian modelling, early warning system, outbreak prediction

## Abstract

Leptospirosis is a zoonotic disease with a high burden in Latin America, including northeastern Argentina, where flooding events linked to El Niño are associated with leptospirosis outbreaks. The aim of this study was to evaluate the value of using hydrometeorological indicators to predict leptospirosis outbreaks in this region. We quantified the effects of El Niño, precipitation, and river height on leptospirosis risk in Santa Fe and Entre Ríos provinces between 2009 and 2020, using a Bayesian modelling framework. Based on several goodness of fit statistics, we selected candidate models using a long-lead El Niño 3.4 index and shorter lead local climate variables. We then tested predictive performance to detect leptospirosis outbreaks using a two-stage early warning approach. Three-month lagged Niño 3.4 index and one-month lagged precipitation and river height were positively associated with an increase in leptospirosis cases in both provinces. El Niño models correctly detected 89% of outbreaks, while short-lead local models gave similar detection rates with a lower number of false positives. Our results show that climatic events are strong drivers of leptospirosis incidence in northeastern Argentina. Therefore, a leptospirosis outbreak prediction tool driven by hydrometeorological indicators could form part of an early warning and response system in the region.

## 1. Introduction

Leptospirosis is a major public health threat, which affects roughly 1.03 million people per year across the globe. Vulnerable populations, such as urban slum dwellers, are particularly affected [1]. Despite its global distribution and high incidence, leptospirosis continues to be considered a neglected tropical disease [2]. Leptospirosis is especially prevalent in tropical and subtropical regions, with the highest burden among low- and middle-income countries [1]. Between 10% and 30% of infections manifest as a febrile syndrome, which, in the absence of treatment, progresses to kidney failure and pulmonary haemorrhage in roughly 10% of clinical cases, resulting in approximately 59 000 fatal infections per year [1,3].

Many mammalian species are carriers of *Leptospira* bacteria, with rodents considered to be the main reservoir of disease in humans [4]. Bacteria are released into the environment via urine, contaminating soil and water bodies [5]. Human exposure occurs after contact with a contaminated environment or animals. Risk factors include occupation (such as farming), inadequate sanitation and housing infrastructure, and recreational activities [3,4].

Heavy rainfall and flooding events are often associated with outbreaks of leptospirosis [4,6]. These events can cause increased environmental exposure to bacteria via contaminated flood water, displacement of rodent populations, as well as damage and contamination of water and sanitation infrastructure [7]. Furthermore, periods of abundant rainfall produce suitable conditions for bacterial survival and rodent proliferation [5,8].

Latin American countries with a tropical and subtropical climate have a high burden of leptospirosis and outbreaks are commonly linked to extreme climatic events [1,9]. In Argentina, *Leptospira* infections happen in the northeastern and central provinces, namely Buenos Aires, Santa Fe and Entre Ríos [10]. Outbreaks occur during the rainy season, which spans from the late spring until early autumn (November–April), and coincide with heavy rainfall and flooding events [11].

El Niño-Southern Oscillation (ENSO) is a global climate phenomenon that influences temperatures and precipitation across the world. It arises from changes in the sea surface temperature (SST) and atmospheric pressure between the western and eastern Pacific Ocean [12]. The ENSO cycle has two distinct phases: the El Niño phase is characterized by positive SST anomalies, while negative anomalies are found during La Niña years [12]. In northeastern Argentina, the El Niño phase tends to lead to an increase in the frequency and intensity of extreme precipitation events [13].

**Figure 1.**
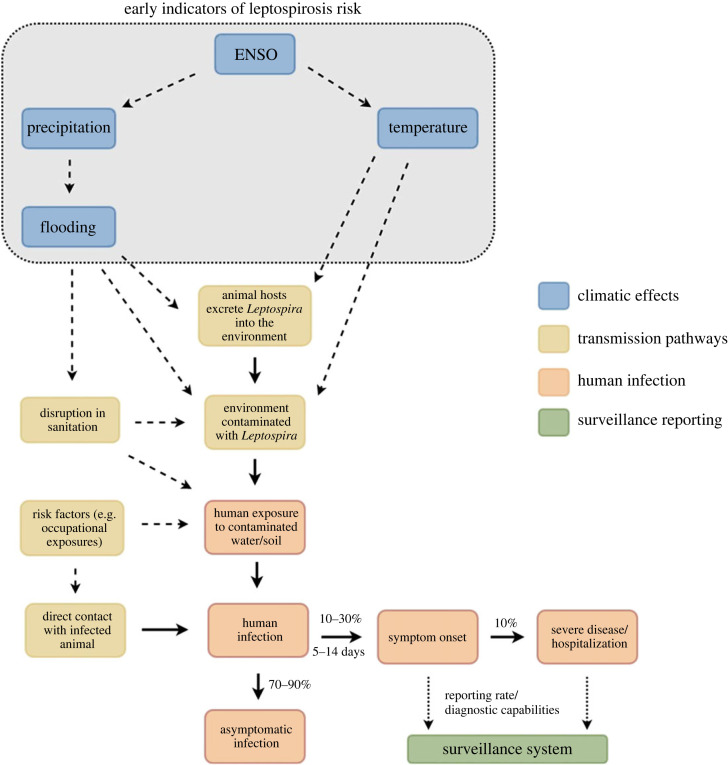
Schematic representation of the transmission and reporting of leptospirosis. *Leptospira* infection in humans can be direct (resulting from direct contact with an infected animal) or indirect (via an environment contaminated by *Leptospira* bacteria). Meteorological variables (including precipitation and temperature) can influence environmental contamination. Once humans have become infected, this usually results in asymptomatic infection. However, around 10–30% of infections result in disease. The majority of those who do experience symptoms have a mild self-limiting disease, although in a small percentage of people (approximately 10%), clinical disease can be severe and result in hospitalization. Depending on the clinical and laboratory capabilities, a proportion of cases will be reported in the surveillance system. Early indicators of leptospirosis risk could feed into a climate-based EWS to produce short- and medium-term forecasts of leptospirosis risk.

Currently, response to leptospirosis outbreaks in Argentina relies on passive surveillance of cases, with delays associated with symptom onset, health-seeking behaviour and laboratory testing (figure 1) [10]. Early warning systems (EWS) can help provide advanced warning of an outbreak, so that interventions can be deployed in a timely manner.

Climate change is expected to increase the frequency and intensity of extreme weather events, which could increase the number and magnitude of leptospirosis outbreaks and other climate-sensitive diseases [6]. Previous mathematical and statistical models in this region have shown promising results that suggest it may be possible to develop an EWS for leptospirosis [11,14,15]. Building on these efforts, this study aims to better characterize the effect of hydrometeorological variables on leptospirosis cases in Santa Fe and Entre Ríos, two provinces in northeastern Argentina. Additionally, this study aims to establish a set of predictive models which could contribute to an EWS, based on readily available climate data, including local weather station data and SST measurements in the Pacific Ocean.

## 2. Methods

### 2.1. Health and population data

**Figure 2.**
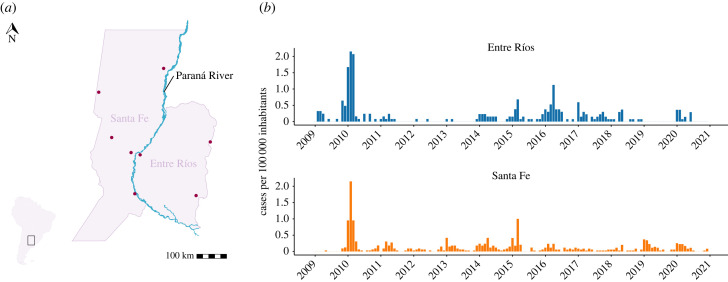
Leptospirosis cases in Entre Ríos and Santa Fe between January 2009 and December 2020. (*a*) Location of Entre Ríos and Santa Fe. Paraná River, one of the most important water bodies of South America, flows in the limit between the provinces. Meteorological information was obtained from stations belonging to the National Meteorological Service (points on the map). (*b*) Number of confirmed leptospirosis cases per 100 000 inhabitants per month recorded in each province and reported to the SIVILA system.

Confirmed leptospirosis cases between January 2009 and December 2020 reported to the National Epidemiological Surveillance System (SIVILA) were provided by the Directorate for Health Promotion and Prevention, Ministry of Health of the Santa Fe province, and the Epidemiology Division of the Entre Ríos province. In the study period, there were 282 confirmed cases in Entre Ríos and 586 in Santa Fe (figure 2*a*,*b*).

Annual population projections per province between 2010 and 2020 were available from the National Institute for Census and Statistics (INDEC) [16]. We used linear regression to extrapolate the population size in 2009.

### 2.2. Hydrometeorological data

The monthly Niño 3.4 index (anomalies in SST in the Niño 3.4 region of the Pacific Ocean; electronic supplementary material, figure S1) from 2008 to 2020 was obtained from the National Oceanic and Atmospheric Administration [17]. Additionally, we used daily precipitation (mm) between January 2008 and December 2020 from eight weather stations (figure 2*a*) [18]. We aggregated daily data to monthly means (mm day^−1^) and then computed population-weighted summaries for each province. Daily Paraná River height (m) records from Paraná City and Santa Fe City stations were collected by the Naval Prefecture and published by the National Institute of Water [19]. Data were available between 2008 and 2020 and were averaged to monthly means (m day^−1^).

### 2.3. Modelling framework

We fitted a series of Bayesian generalized linear mixed models independently for each province. We assumed that monthly leptospirosis case counts followed a negative binomial distribution and included random effects to account for unobserved seasonality and interannual variability.

Model fitting was based on the work done by Lowe *et al*. and Colón-González *et al*. [20–22]. We started by building an intercept-only model and then increased model complexity by incorporating seasonal and interannual random effects. Next, we included all possible combinations of variables and evaluated their performance using goodness of fit (GOF) statistics described below. We then evaluated the ability of candidate models to produce out-of-sample predictions. We fitted models and estimated marginal posterior predictive distributions of both random and fixed parameters using the integrated nested Laplace approximation [23]. Details of model structure and development can be found in the electronic supplementary material.

Since ENSO acts as a driver of precipitation anomalies in the region, we explored the role of ENSO independently from local conditions (precipitation and river height). ENSO models included SST anomalies lagged from 1 to 12 months, while local models were built with combinations of precipitation and river height lagged from 1 to 5 months. In total, we fitted 49 models for each province: one intercept-only model, one random effects-only model, 12 ENSO models (lags of 1 to 12 months) and 35 local models (combinations of river height and precipitation, with lags of 1–5 months each).

### 2.4. Model selection

A subset of models for each province was selected based on multiple measures of GOF: (i) deviance information criterion (DIC), (ii) likelihood ratio *R*^2^ and (iii) visual inspection of observed versus fitted case counts. Based on these criteria, we selected one ENSO model and one local model for each province (electronic supplementary material).

### 2.5. Model predictive check

Once we selected the candidate models, we simulated out-of-sample predictions and evaluated their performance compared to a reference model. Since there is no current EWS in place, we used the least informative model as a reference, which included seasonal random effects only. We left 12 consecutive months out of the training dataset starting with January 2009 and moved one month forward for each subsequent run (144 runs per model). For each run, we drew 1000 samples from the posterior marginal distribution of the parameters and used them to compute posterior predictive counts from a negative binomial distribution. To assess model performance, we used the continuous rank probability score (CRPS) and the continuous rank probability skill score (CRPSS), which is a relative measure of model performance, compared to a reference model (electronic supplementary material).

### 2.6. Outbreak detection evaluation

We then assessed the ability of the candidate models to predict outbreaks. We defined an outbreak threshold as the moving 75th percentile of the observed case counts and computed an outbreak probability by counting the number of samples that exceeded this threshold. We used these outputs to create receiving operator characteristic (ROC) curves to determine a probability trigger threshold, defined as the point on the curve that maximized the hit rate (HR), i.e. sensitivity, and minimized the false alarm rate (FAR), i.e. 1 − sensitivity. We compared performance in outbreak detection in each of the candidate models to the reference model (monthly random effects-only model), using the area under the ROC curve (AUC), as well as the HR and FAR (electronic supplementary material).

## 3. Results

### 3.1. Data description

Between 2009 and 2020, there were 282 confirmed leptospirosis cases in Entre Ríos and 586 in Santa Fe. We observed sporadic outbreaks, with the highest being in 2010, 2014, 2015 and 2016 (figure 2*b*). The mean annual number of cases ranged from 0 to 6.69 cases per 100 000 inhabitants in Entre Ríos and from 0.25 to 4.85 cases per 100 000 inhabitants in Santa Fe (electronic supplementary material, table S1). Most leptospirosis cases occurred during and after the wet season (November–April) in both provinces. Heavy precipitation and flooding events happened in 2010 and 2016, which coincided with a sharp rise in the number of reported leptospirosis cases. Additionally, outbreaks occurred during El Niño phases, while, conversely, very few cases were recorded in La Niña years, such as 2011 and 2013 (electronic supplementary material, figure S2).

### 3.2. Model selection

**Figure 3.**
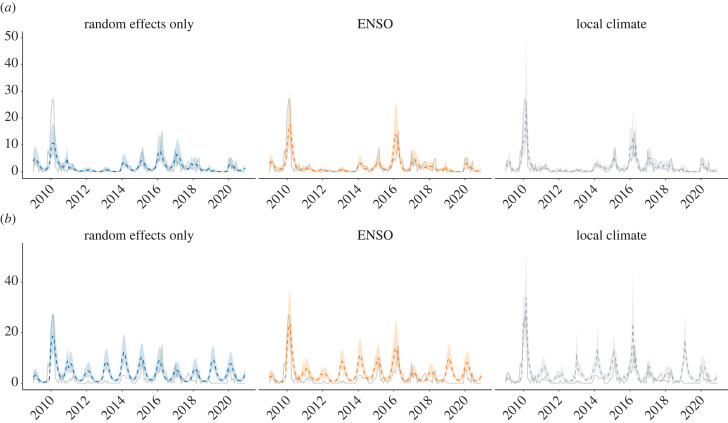
Fitted versus observed leptospirosis cases per month. Posterior median (dashed lines) and posterior 95% credible intervals (shaded area) for number of leptospirosis cases per month in (*a*) Entre Ríos and (*b*) Santa Fe between January 2009 and December 2020. Observed values (solid line) were recorded by the national surveillance system. Estimates are presented for the random effects-only model, and the best-fitting ENSO and local climate models (dashed lines).

Hydrometeorological covariates, i.e. the Niño 3.4 index, Paraná River height and precipitation, improved model GOF in each province, compared to their respective random effects-only models. The Niño 3.4 index with a three-month lag had the best model fit compared to the other lags. Overall, this model captured 58% of the variability in the data from Entre Ríos and 61% from Santa Fe. Including the Niño 3.4 index captured an additional 10% of the variability in the data from Entre Ríos and an additional 3% in Santa Fe, compared to the random effects-only models (table 1). Noticeably, the effect of ENSO in Santa Fe was weaker than in Entre Ríos (electronic supplementary material, figure S3). This difference is more evident in the time series of observed versus fitted values (figure 3). In Entre Ríos, the fitted model values were able to capture the peaks in 2010, 2014, 2016 and 2017. However, in Santa Fe, the model overestimated cases during periods of low incidence (i.e. 2013 and 2019).

**Table 1.** Measures of GOF in candidate models. Models were formulated with increasing complexity and compared to a non-informative intercept-only model. ENSO models in both provinces were built using the Niño 3.4 index lagged by three months. Local models comprised river height and precipitation with one-month lags in both provinces. REs, random effects; DIC, deviance information criterion; *R*^2^_(NULL)_, likelihood ratio *R*^2^ with reference to the intercept-only model; *R*^2^_(REs)_, likelihood ratio *R*^2^ with reference to the random effects-only model.

	DIC	*R*^2^_(NULL)_	*R*^2^_(REs)_
*Entre Ríos*			
intercept only	528	–	–
RE only model	446	0.53	–
ENSO model	433	0.58	0.1
local model	437	0.57	0.08
*Santa Fe*			
intercept only	725	–	–
RE only model	621	0.6	–
ENSO model	618	0.61	0.03
local model	605	0.64	0.12

Both precipitation and river height lagged by one month were good predictors in Entre Ríos and Santa Fe. Compared to the random effects-only model, including local climate captured an additional 8% of the variability in the data from Entre Ríos and 12% from Santa Fe (table 1). In Entre Ríos, river height had a higher mean posterior effect size than precipitation, although their 95% credible intervals overlapped (electronic supplementary material, figure S3).

### 3.3. Model predictive checks and outbreak detection

Hydrometeorological candidate models outperformed their respective reference models in out-of-sample predictions (table 2). Performance was higher in Entre Ríos than in Santa Fe. ENSO models showed an improvement in the CRPSS of 39% in Entre Ríos and 9% in Santa Fe, compared to the reference model, which only included seasonal random effects. Furthermore, local climate models performed better than the reference models in both provinces and better than the ENSO model in Santa Fe (electronic supplementary material, tables S2 and S3, and figure S4).

**Table 2.** Area under the ROC curve (AUC) to show overall model performance. ENSO models in both provinces were formulated using the Niño 3.4 index lagged by three months. Local models comprised river height and precipitation with one-month lags in both provinces. Confidence intervals computed from 2000 bootstrap samples.

	AUC (95% CI)
*Entre Ríos*	
seasonal effects only (reference)	0.77 (0.64–0.9)
ENSO model	0.94 (0.89–1)
local model	0.95 (0.89–1)
*Santa Fe*	
seasonal effects only (reference)	0.81 (0.73–0.9)
ENSO model	0.88 (0.82–0.95)
local model	0.92 (0.87–0.97)

**Figure 4.**
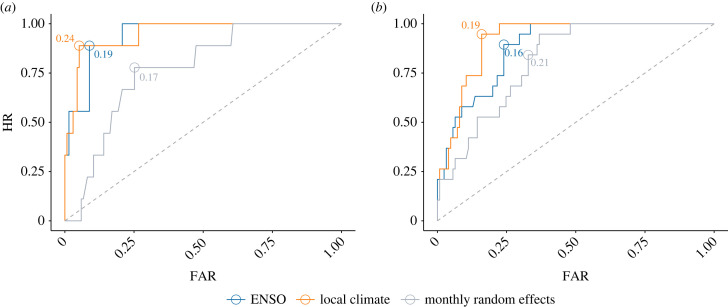
ROC curves. Performance of candidate models in detecting outbreaks in (*a*) Entre Ríos and (*b*) Santa Fe. The points for which the probability thresholds correspond to the optimal balance between HR and FAR, i.e. closest to the point of perfect classification (0, 1), are circled and the values are displayed on the graph. The diagonal dashed lines represent the point at which the HR equals the FAR, indicating a model where the performance is no better than a coin toss.

In Entre Ríos, both candidate models showed high predictive performance levels, with AUC values of 0.94 for the ENSO model and 0.95 for the local climate model (figure 4). Additionally, HR values were high in both provinces, with the local climate models reducing FAR levels in comparison to the ENSO models (electronic supplementary material, tables S2 and S3).

### 3.4. Model translation into an early warning system

**Figure 5.**
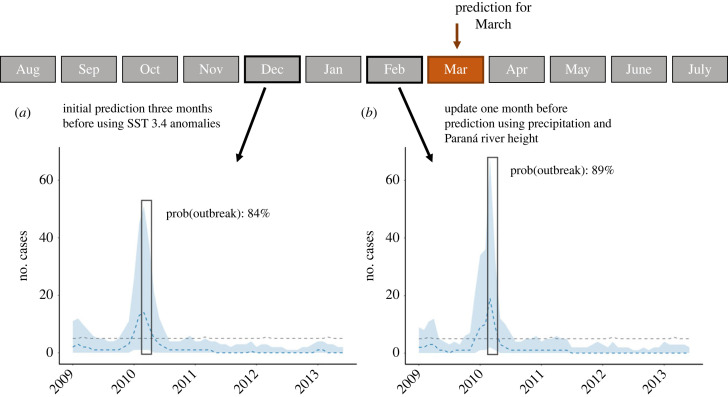
Schematic timeline demonstrating the two-stage computation of outbreak probability for March 2010 in Entre Ríos. Public health officials with access to surveillance reports and data from the national meteorological service would compute the probability of an outbreak ahead of its occurrence. In this example, (*a*) a first prediction with the Niño 3.4 index from December 2009 was computed after leaving data between January and December 2010 out of sample. Then, (*b*) a second prediction with precipitation and river height measurements from February 2010 was computed by excluding data from March 2010 until February 2011. Grey dashed line: outbreak threshold computed as the 75th percentile of cases observed in the target month excluding the number of cases in the year of interest.

We propose a two-stage forecasting approach for leptospirosis. Public health users would produce an early-stage outbreak probability using the Niño 3.4 index three months ahead of the target month and later update the outbreak probability with precipitation and river height information from the month prior to the outbreak. As an example, the ENSO model computed an outbreak probability for March 2010 of 84% in Entre Ríos. Then, the short-lead local model increased that percentage to 89% (figure 5).

## 4. Discussion

In this study, we present statistical models that were able to quantify the impact of hydrometeorological variables on the number of leptospirosis cases reported to the surveillance system and were used to compute out-of-sample predictions with high performance. This work contributes to the increasing literature demonstrating the relevance of Bayesian hierarchical mixed models for making robust predictions for use in public health responses [24].

During El Niño years, northeastern Argentina experiences an average increase in the frequency and intensity of extreme rainfall, which can result in flooding events [25]. Our results show that leptospirosis cases reported to the surveillance system are associated with changes in SST in the Niño region 3.4, which serves as an ENSO phase indicator [26]. Notably, we found that the ENSO models performed better in Entre Ríos than in Santa Fe. This may be partially explained by differences in the geomorphological configuration. While the Paraná river has ravines of different sizes on the Entre Ríos coast, there is a flat and smooth gradient descending towards the alluvial plain on the Santa Fe side, creating differences in the association between river levels and flooding events [27]. Additionally, the presence of less important water courses with different sensitivities to ENSO, such as the Salado and Uruguay rivers, may have affected model performance.

At the local level, the climate models performed well in fitting leptospirosis cases in both provinces, which coincides with previous findings in the literature [4,7]. The effect of river height was greater than that of precipitation in Entre Ríos, although their credible intervals overlapped. In Santa Fe, however, we did not see differences between the effects of local conditions.

We tested the out-of-sample predictive ability of our models by sequentially leaving 12 months out of the sample. Since there is no current EWS in place, we used the least informative model as a reference, which included seasonal random effects only. In both provinces, the candidate models outperformed the reference model, particularly in Entre Ríos.

Given these findings, we propose a two-stage prediction approach for Entre Ríos and Santa Fe provinces, comprising an initial forecast using the ENSO model with a lead time of three months, followed by the local climate model one month prior to the prediction target. Although local climate models did not show a large increase in predictive performance in comparison to the ENSO models, they showed a reduction in their FARs. A high outbreak probability for the upcoming month would trigger the deployment of different prevention strategies such as pre-exposure prophylaxis, identifying populations at risk and increasing awareness in the local medical community. To our knowledge, only one study proposed a prediction model for leptospirosis in New Caledonia [28].

Until recently, development of climate-based EWS for infectious diseases has been mostly done in academic settings. There are many challenges in the implementation of EWS, including lack of historical and real-time data, as well as coarse spatial and temporal resolutions [29]. Moreover, to translate prediction models and code into usable, automated tools, funding for software development and maintenance is necessary. To implement a leptospirosis EWS, joint efforts between local stakeholders, national public health agencies and researchers would be required [24]. Our prediction models could be delivered in the form of executable packages or dashboards, with functions that allow users to input and explore climate data, and compute outbreak probabilities. Capacity building would be another important component of the implementation process as users need to be able to manage data and interpret model outputs using their local resources.

Although this study showcases the potential of modelling tools for informing public health action, there are several limitations. First, spatially aggregated data mask the effects of hydrometeorological drivers of disease at finer spatial scales. A recent analysis of the spatial distribution of leptospirosis suitability in Santa Fe province has shown that there is marked difference with elevation, urban and suburban structure, and environmental conditions [30]. Most leptospirosis cases were reported in the largest cities and in some of them there are other water bodies, such as the Salado River in Santa Fe. Moreover, working with surveillance data produced from mandatory reports can be challenging due to reporting delays, data sparsity and protection, and limited information regarding sociodemographic factors, which may act as additional explanatory variables. Surveillance also depends on presentation of symptoms, with mildly symptomatic and asymptomatic cases being largely underreported. However, predicting the risk of symptomatic and severe cases would provide great added value for planning and prevention through public health strategies.

Data sparsity also affects the quality of hydrometeorological records. Flooding events have differences in the degree of the damage caused and their duration. The severe flood caused by the Paraná River in 2016, for example, left areas covered by water for several months, during which the victims had to undergo evacuation [31,32]. During this period, there were more intensive preventive campaigns than in other years, and this is likely the reason we do not see such a large increase in cases, even though the climatic conditions were suitable [11]. Furthermore, grouping data at the province level assumes that there is an even distribution of leptospirosis cases in both provinces, when case distribution is highly heterogeneous [11]. The ability of the surveillance system to capture cases varies across the region due to differences in access to healthcare and socio-economic status, with more rural populations and those from a lower socio-economic background likely to be more underreported. The results from this study demonstrate the benefits that could be gained from further investment into leptospirosis surveillance in the future. Despite these limitations, we found that the role of climate appears to be associated with cases even over a large geographical area, demonstrating the potential utility of an EWS.

In the future, longer lead times for forecasts may be possible. The local meteorological service and the National Water Institute produce forecasts with lead times of up to three months for precipitation and river height, respectively [18,19]. These forecasts are released to the public as bulletins, although currently the information they report is not consistent, as the time of reporting and the stations included within the bulletin vary. In the context of an interdisciplinary collaboration with direct access to data, it may be possible to produce outbreak forecasts with a longer lead time.

The proposed two-stage modelling approach is a first step towards the creation of a high-performance prediction tool for a leptospirosis EWS in northeastern Argentina. This would allow public health services to have advanced warning of an outbreak, allowing time for the implementation of public health preventative measures. This will become more important in the future with climate change expected to increase the frequency and intensity of flooding events and, in turn, outbreaks of leptospirosis and other climate-sensitive diseases.

## Data Availability

The code and data used to produce the analysis can be found on https://github.com/martin-lotto-batista/lepto-argentina, archived in a permanent repository: https://zenodo.org/record/7865639 [33]. Additional details on the methodology can be found in the electronic supplementary material [34].
